# Disparate In Vivo Efficacy of FTY720 in Xenograft Models of Philadelphia Positive and Negative B-lineage Acute Lymphoblastic Leukemia

**DOI:** 10.1371/journal.pone.0036429

**Published:** 2012-05-03

**Authors:** Craig T. Wallington-Beddoe, Anthony S. Don, John Hewson, Qiao Qiao, Rachael A. Papa, Richard B. Lock, Kenneth F. Bradstock, Linda J. Bendall

**Affiliations:** 1 Westmead Institute for Cancer Research, Westmead Millennium Institute, The University of Sydney, Sydney, Australia; 2 Lowy Cancer Research Centre, Prince of Wales Clinical School, Faculty of Medicine, University of New South Wales, Sydney, Australia; 3 Children's Cancer Institute Australia for Medical Research, Lowy Cancer Research Centre, University of New South Wales, Sydney, Australia; 4 Hematology Department, Westmead Hospital, Westmead NSW, Australia; Texas A&M University, United States of America

## Abstract

Most patients with acute lymphoblastic leukemia (ALL) respond well to standard chemotherapy-based treatments. However a significant proportion of patients, particularly adult patients, relapse with the majority dying of leukemia. FTY720 is an immunosuppressive drug that was recently approved for the treatment of multiple sclerosis and is currently under pre-clinical investigation as a therapy for a number of hematological malignancies. Using human ALL xenografts in NOD/SCIDγc^−/−^ mice, we show for the first time that three Ph^+^ human ALL xenografts responded to FTY720 with an 80±12% (p = 0.048) reduction in overall disease when treatment was commenced early. In contrast, treatment of mice with FTY720 did not result in reduced leukemia compared to controls using four separate human Ph^−^ ALL xenografts. Although FTY720 reactivated PP2A *in vitro*, this reactivation was not required for death of Ph^−^ ALL cells. The plasma levels of FTY720 achieved in the mice were in the high nanomolar range. However, the response seen in the Ph^+^ ALL xenografts when treatment was initiated early implies that *in vivo* efficacy may be obtained with substantially lower drug concentrations than those required *in vitro*. Our data suggest that while FTY720 may have potential as a treatment for Ph^+^ ALL it will not be a useful agent for the treatment of Ph^−^ B-ALL.

## Introduction

Acute lymphoblastic leukemia (ALL) represents approximately a quarter of all childhood cancers, and a similar proportion of cases of acute leukemia in younger adults. Although the treatment of childhood ALL is one of the success stories of modern oncology, treatment protocols remain imperfect [1]. Approximately 15% of children and the majority of adults diagnosed with ALL relapse following treatment. The overall survival of the 60% of adults who relapse following treatment is only 7% at 5 years [2], [3]. Overall, once relapse occurs, the success of any further treatment, including hematopoietic stem cell transplantation, is poor.

FTY720 is an immunosuppressive drug recently approved for the treatment of multiple sclerosis [4]. Once phosphorylated by sphingosine kinases, phosphorylated FTY720 (FTY720-P) down regulates four of the five sphingosine 1-phosphate (S1P) receptors, trapping lymphocytes in secondary lymphoid organs [5]. More recently, FTY720 has been investigated for the treatment of malignancies and has documented *in vitro* and/or pre-clinical activity against a number of hematological disorders including T-cell acute lymphoblastic leukemia (T-ALL), multiple myeloma, chronic lymphocytic leukemia (CLL), mantle cell lymphoma (MCL), acute myeloid leukemia (AML) with c-kit mutations, mouse models of chronic myeloid leukemia (CML) and Ph^+^ (Philadelphia chromosome positive) ALL, Ph^−^ ALL and NK cell leukemia [6], [7], [8], [9], [10], [11], [12], [13].

The anti-leukemic efficacy of FTY720 is thought to be due to reactivation of the protein phosphatase type 2A (PP2A), an essential protein serine/threonine phosphatase, the activity of which is reduced in certain malignancies [6]. The involvement of PP2A reactivation in Ph^+^ disease has been well documented with interplay between PP2A and Bcr/Abl being clearly demonstrated [11]. Indeed PP2A activation and caspase-dependent cell death were required for its cytotoxic effect in AML, CML and Ph^+^ ALL [10], [11] whilst caspase-dependence without PP2A activation was recently reported for NK cell leukemia [13]. However we, and others, have reported caspase-independent death mechanisms of FTY720, suggesting that the mechanism of action of FTY720 in malignant cell killing is varied and still unclear [8], [9], [12]. Regardless of the mechanism of cell death, the IC_50_ values have been similar between studies, ranging from 2.4 to 12 µM (Table 1). Study of the *in vivo* efficacy of FTY720 for the treatment of CLL, MCL, AML, CML, Ph^+^ ALL and NK cell leukemia demonstrated increased survival of mice and/or reduced leukemic cell burden [8], [9], [10], [11], [13].

**Table 1:** Studies of FTY720 in hematological malignancies.

Study	Cell Type	Caspase-dependent	PP2A-dependent	IC_50_ range (µM)	In Vivo	
					Daily Dose	Increased Survival
Matsuoka et al 2003.	T-ALL	Yes	Yes	ND	ND	ND
Yasui et al 2005.	Myeloma	Yes	ND	3.6–9.7	ND	ND
Wallington-Beddoe et al 2011.	Ph^+^ & Ph^−^ ALL	No	No	5.3–7.9	ND	ND
Neviani et al 2007.	CML, Ph^+^ ALL	Yes	Yes	ND	10 mg/kg	Yes (32D & BaF3)
Liu et al 2008.	CLL, Burkitt's Lymphoma	No (CLL), No (Raji), Yes (Ramos)	Yes (CLL), ND (Raji), Yes (Ramos)	4→10 (CLL), 4→10 (Raji, Ramos)	5 mg/kg	ND (CLL, Ramos), Yes (Raji)
Liu et al 2010.	MCL	No	ND	5–12	5 mg/kg	Yes (JeKo)
Roberts et al 2010.	AML	Yes	Yes	2.4–7.5 (48 h)	10 mg/kg	Yes (FDC-P1 with activated c-kit)

ND - Not determined. *In vitro* IC_50_ values were assessed at 24 hours unless otherwise indicated. FTY720 was administered by the intra-peritoneal route in all *in vivo* studies. Cell lines used for *in vivo* studies are indicated.

We have previously reported the *in vitro* efficacy of FTY720 in Ph^−^ ALL [12]. Here, consistent with reports by others in mouse models of Ph^+^ ALL [11], we show that FTY720 was effective *in vivo* in a human xenograft model of Ph^+^ ALL. On the other hand, we found that FTY720 had no therapeutic effect *in vivo* against Ph^−^ ALL. This disparity in the *in vivo* response occurred despite Ph^+^ and Ph^−^ ALL cells demonstrating similar *in vitro* sensitivities to FTY720. In some Ph^−^ ALL xenografts FTY720 appeared to exacerbate the disease, suggesting that clinical trials of FTY720 in Ph^−^ ALL are unlikely to succeed.

## Materials and Methods

### Cells and Reagents

Leukemic blasts were obtained from 4 ALL patients with written informed consent, or in the case of minors from the parents of patients, and institutional ethics committee approval from the Sydney West Area Health Service Human Ethics Committee (Approval No. HREC/2009/8/4.1 3028), while xenografts ALL-3, ALL-55 and ALL-56 were previously established. The clinical details of some patient samples have been previously published but information on all patient samples are summarized in Table S1 [14], [15], [16], [17]. Xenografts were established in NOD/SCID mice from mononuclear cells as described previously [14]. The phosphatase inhibitor okadaic acid was purchased from Sigma-Aldrich (St Louis, MO) and FTY720 from Selleck Chemicals (Houston, TX).

### Assessment of In Vivo FTY720 Efficacy

This study was carried out in strict accordance with the recommendations in the National Health and Medical Research Council Guidelines and Policies to Promote the Wellbeing of Animals Used for Scientific Purposes and the Australian Code of Practice for the care and use of animals for scientific purposes. Protocols were approved by the Sydney West Area Health Service Animal Ethics Committee (Approval No. 5049.08-09) and The University of New South Wales Animal Care and Ethics Committee (Approval No. 09/130A).

Groups of NOD.Cg-Prkdcscid Il2rgtm1Wjl/SzJ (NOD/SCIDγc^−/−^) mice were engrafted with 2–5×10^6^ ALL cells by tail vein injection. Peripheral blood was collected weekly from the tail vein of all mice for the monitoring of ALL. FTY720, prepared in 2% ethanol (experiments using ALL-3) or saline (all other experiments), was administered by intra-peritoneal (IP) injection, except where gavage was indicated. For the early disease model, treatment commenced within a week of cell transfer and for the advanced disease model, when 1% ALL was detected in the blood. All mice were treated for 3 weeks unless otherwise indicated, with mice engrafted with xenograft ALL-3 receiving drug 6 days a week, while all others received treatment daily. Animals from xenografts 1345, 2070, 1999, 0398, ALL-55 and ALL-56 were sacrificed after 21 days of treatment and disease assessed in the peripheral blood, bone marrow and spleen by flow cytometry and in the liver by histology. Single cell suspensions of blood, bone marrow and spleen were prepared and red cells removed by lysis where required. Total leukemia burden was calculated by totaling the number of ALL cells in the bone marrow, blood and spleen. The calculation of total ALL cells in the bone marrow was based on the accepted standard that the marrow from one femur represents 5.8% of the total bone marrow. Total ALL cells in the blood was based on the total blood volume being 80 µL/g of mouse.

### Flow Cytometry

Cell viability was measured as previously described [12] after a 16-hour exposure to FTY720. Cells were labelled with propidium iodide and annexin V-FITC (BD Biosciences, San Jose CA) according to the manufacturer's instructions, with negative cells considered viable.

Cells from mice were stained with anti-human CD19PE or anti-human CD45APC and anti-murine CD45FITC (BD Biosciences and Invitrogen, Carlsbad CA) for 10 or 30 minutes according to the manufacturers' instructions, and as previously described [14], [17]. All cells were analysed using a FACSCanto flow cytometer (BD Biosciences).

### PP2A Activity Assay

Cells were lysed in a low-detergent lysis buffer (1% Nonidet P-40, 10 mM HEPES, 150 mM NaCl, 10% glycerol, 1 mM PMSF, 5 mM benzamidine and 10 µg/mL leupeptin). PP2A phosphatase activity was determined using the malachite green-phosphate complex assay as described by the manufacturer (Millipore, Billerica MA) using a PP2A-specific reaction buffer and 750 µM phosphopeptide substrate. After 10 minutes incubation at 30°C, malachite dye was added and free phosphate measured by optical density at 620 nm using a Wallac 1420 Multilabel Counter (PerkinElmer, Turku Finland).

### Measurement of FTY720 in plasma

Blood was drawn by tail vein bleeding and immediately transferred to ice. A 50 µL aliquot was centrifuged at 1500 rpm for 10 min to pellet cells, and 20 µL of plasma was added to 380 µL ice-cold methanol. Extracts were spiked with 20 pmoles C17 sphingosine and C17 S1P (Avanti Polar Lipids, Alabaster, AL), which act as the internal standards for FTY720 and FTY720-phosphate (FTY720-P), respectively. Extracts were then vortexed, sonicated for 30 seconds in an ice bath, and centrifuged for 20 min at 14,000 rpm, at 4°C. Supernatants were transferred to 5 mL glass tubes, and the pellets were re-extracted as above with 600 mL ice-cold 80% methanol/20% water (v/v). The supernatants from both extraction steps were combined, dried in a SpeediVac, resuspended in 200 µL 80% methanol/0.1% formic acid (v/v), and stored at −20°C for quantification of FTY720 and FTY720-P using liquid chromatography-tandem mass spectrometry (LC-MS/MS), as described previously [18].

### Statistical Analysis

Comparisons between two groups were performed using the Student's t test and between multiple groups using two-way ANOVA with Bonferroni post-test. Comparison of the response of Ph^−^ and Ph^+^ ALL xenografts was done using a Fischer's exact test.

## Results

### FTY720 Did Not Reduce the Progression of a Ph^−^ ALL in Vivo Using Multiple Treatment Dosing Schedules

**Figure 1:**
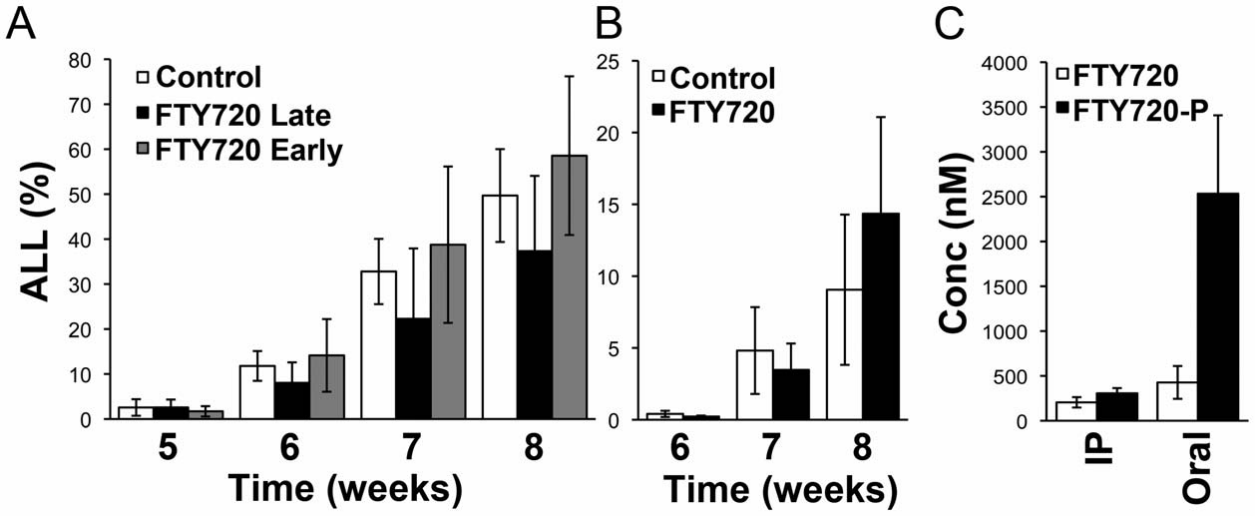
FTY720 does not reduce disease burden in a xenograft model of advanced human ALL. (A) Groups of 7–8 NOD/SCIDγc^−/−^ mice were engrafted with xenograft ALL-3 and treated with vehicle control or FTY720 by IP injection 6 days a week for 3 weeks. The early disease arm received FTY720 at 5 mg/kg/day 6 days a week commencing on day 4 post engraftment, and the advanced disease arm at 10 mg/kg/day commencing in week 5. The mean ± SD of the percentage of ALL cells in the blood is indicated. (B) Groups of 4 mice were engrafted with ALL-3 and treated with 25 mg/kg/day of FTY720 or vehicle control by gavage, commencing in week 6 and continuing for 2 weeks. The mean ± SD of the percentage of ALL cells in the blood is indicated. (C) Plasma concentrations of FTY720 and FTY720-P were determined 2 h after IP administration and 6 h after oral administration of FTY720.

FTY720 has been reported to induce cell death in ALL cells *in vitro* [12] and inhibit the development of ALL in the murine cell line BaF3 transduced with Bcr/Abl in immuno-compromised mice [11]. Here we examined the effect of FTY720 on Ph^−^ human ALL xenografts in NOD/SCID mice. In an initial experiment using xenograft ALL-3, we compared an advanced disease model, where treatment was not commenced until disease was clearly detectable in the blood, with an early disease model where treatment commenced only 4 days post engraftment. Mice were administered vehicle control or FTY720 by IP injection at 5 mg/kg/day in the early disease arm or 10 mg/kg/day in the late disease arm. Mice were treated for a total of 18 days and the level of ALL in the blood assessed weekly. Although a trend towards reduced disease with FTY720 was observed, this did not achieve statistical significance (Fig. 1A). To further escalate plasma concentrations of FTY720, this same xenograft was examined using orally administered FTY720 at 25 mg/kg/day, with treatment commencing when human leukemia cells were first detected in peripheral blood (advanced disease model). Again there was no reduction in disease observed in the FTY720-treated animals (Fig. 1B). Assessment of FTY720 plasma levels revealed mice receiving the higher dose of drug by gavage had higher FTY720 levels (427.1±58 nM) than those receiving FTY720 by the IP route (205±58 nM) (Fig. 1C). Consistent with previous reports, FTY720-P was present at higher levels than the parental drug in the plasma of treated mice [18], [19].

### FTY720 Is Effective in Ph^+^ but Not Ph^−^ ALL Xenografts Using an Early Disease Model

**Figure 2:**
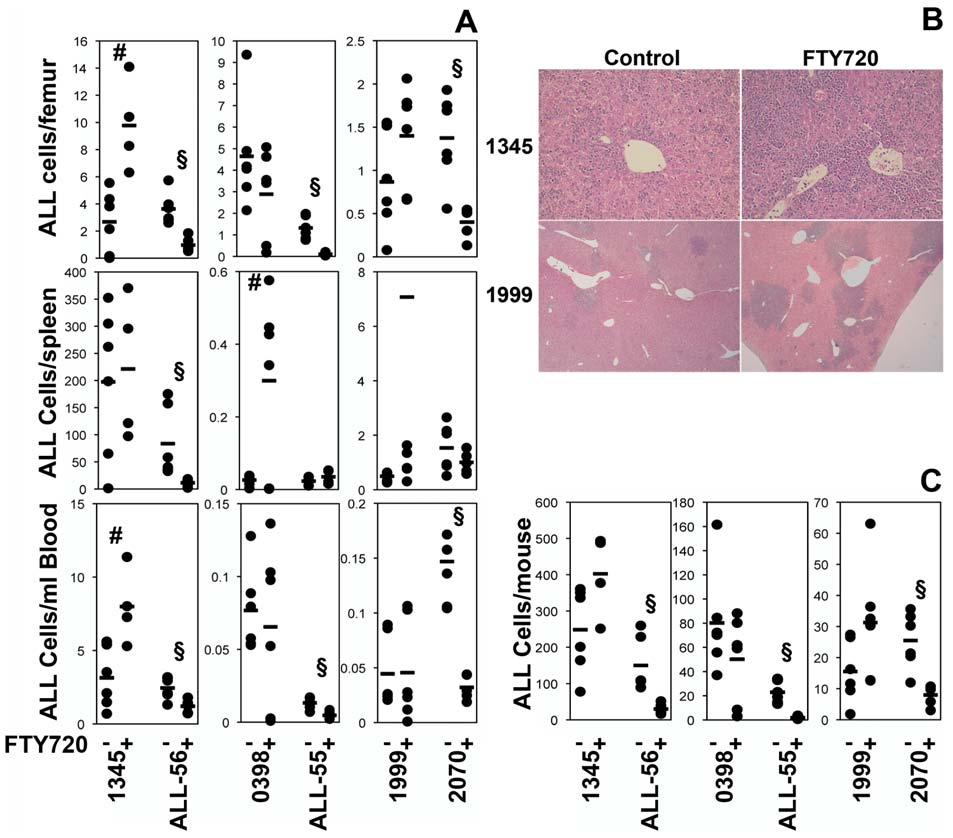
FTY720 does not reduce Ph− ALL in a μodel of early disease. (A) Groups of 6 NOD/SCIDγc^−/−^ μice were engrafted with the indicated human ALL xenografts. After 3 to 7 days, 10 mg/kg (1345, 1999, 2070, ALL-55 and ALL-56) or 5 mg/kg (0398) of FTY720, or 0.9% sodium chloride was administered daily by intra-peritoneal injection for three weeks. Surviving animals were sacrificed at the end of treatment and the level of leukemia analyzed by flow cytometry. The number of ALL cells ×10^6^ in the bone marrow, spleen and blood is reported with each dot representing an individual animal and the bar the mean of the cohort. #p<0.05 indicating increased disease and §p<0.05 reduced disease compared to control. (B) Increased infiltration of the liver following FTY720 treatment. Livers collected at the time of sacrifice from the animals shown in figure 1A were formalin fixed and paraffin embedded. Sections were stained with hematoxylin and eosin and examined by light microscopy. Representative control and FTY720-treated sections are shown for xenografts 1345 and 1999. Xenografts 0398 and 2070 had minimal leukemia in the liver. (C) Total ALL burden was calculated for each animal from the numbers of ALL cells in each compartment (peripheral blood, bone marrow and spleen). The number of ALL cells ×10^9^ is reported with each dot representing an individual animal and the bar the mean of the cohort. #p<0.05 indicating increased disease and §p<0.05 reduced disease compared to control.

To further examine whether FTY720 may be effective when used earlier in the development of the disease, and in light of the possibility that the ALL-3 xenograft may have been resistant to FTY720, we engrafted NOD/SCIDγc^−/−^ mice with three additional human Ph^−^ ALL xenografts (1345, 0398 and 1999). Treatment was commenced on day 3 (xenograft 1345) or 7 (xenografts 0398 and 1999) post engraftment, when ALL cells were not yet detectable in the peripheral blood, and continued for 3 weeks. No significant reduction in disease was observed in any tissue examined at the end of 3 weeks of treatment with FTY720. Indeed, the amount of ALL increased in the bone marrow from 2.7±2.3 to 9.8±3.3×10^6^ cells/femur (3.7 fold, p = 0.004) and blood from 3.1±2.1 to 8.0±2.5×10^6^ cells/mL (2.5 fold, p = 0.01) of mice with xenograft 1345 treated with FTY720 and in the spleens, from 0.03±0.01 to 0.30±0.24×10^6^ cells (11.5 fold, p = 0.02) of mice receiving xenograft 0398 (Fig. 2A). In the experiment using xenograft 1345 there were also two deaths in the FTY720 group prior to the three-week endpoint with very high levels of leukemia in both animals (data not shown). In addition, the livers of mice engrafted with xenografts 1345 and 1999 also showed increased leukemic cell infiltration (Fig. 2B). The dose of FTY720 was reduced from 10 mg/kg to 5 mg/kg in the experiment using xenograft 1999 due to concerns regarding toxicity of the drug, with extreme lethargy noted post injection in preceding xenografts.

To confirm the validity of this model we examined the response of 3 Ph^+^ ALL samples, 2070, ALL-55 and ALL-56. Mice were engrafted and treated as described above with treatment commencing 7 days after the injection of cells. Consistent with reports on murine Ph^+^ ALL [11], FTY720 produced a significant reduction in disease burden in the Ph^+^ ALL xenografts using an early disease model (Fig. 2A). This decrease was most obvious in the blood (reduced from 0.15±0.04 to 0.03±0.00×10^6^/mL (p = 0.0002), 0.01±0.00 to 0.00±0.00×10^6^/mL (p = 0.0009), and 2.45±0.76 to 1.12±0.41×10^6^/mL (p = 0.005) respectively) and the bone marrow (reduced from 1.38±0.51 to 0.41±0.18×10^6^/femur (p = 0.003), 1.32±0.51 to 0.10±0.06×10^6^/femur (p = 0.0002) and 3.63±1.16 to 0.96±0.51×10^6^/femur (p = 0.0004) respectively) with variable responses being noted in the spleens (from 1.53±0.87 to 1.00±0.39×10^6^/spleen (p = n.s.), 0.02±0.01 to 0.04±0.02×10^6^/spleen (p = n.s.) and 83.54±65.04 to 11.39±6.44×10^6^/spleen (p = 0.02) respectively). Overall the Ph^+^ xenografts were reduced from an estimated total disease burden of 25.48±9.06 to 7.97±3.40×10^6^ cells (p = 0.002), 22.85±8.96 to 1.81±1.08×10^6^ cells (p = 0.0002) and from 149.96±74.08 to 29.92±13.36×10^6^ cells (p = 0.003) respectively. The response of Ph^+^ xenografts was significantly different from that observed in Ph^−^ xenografts (p = 0.029).

### FTY720 Kills Ph^−^ ALL Xenograft Cells in Vitro in a PP2A-Independent Manner

**Figure 3:**
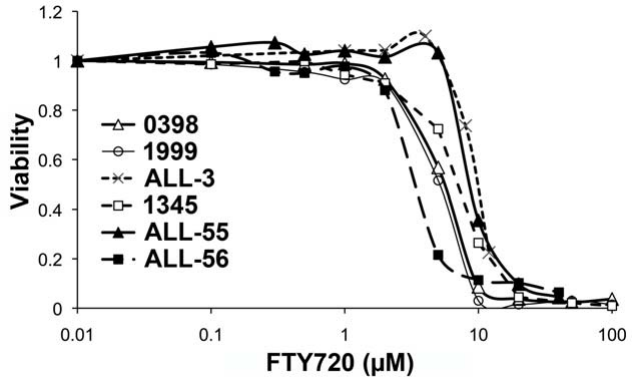
Induction of cell death *in vitro* of the indicated xenograft cells. ALL cells recovered from the spleens of untreated mice were cultured with the indicated concentrations of FTY720 for 16 hours and viability assessed by flow cytometry using annexin V and propidium iodide staining. The viability of cells in control cultures was 42% for xenograft 1345, 90% for xenograft 1999, 93% for xenograft 0398, 51% for xenograft ALL-3, 63% for xenograft ALL-55 and 34% for ALL-56.

**Figure 4:**
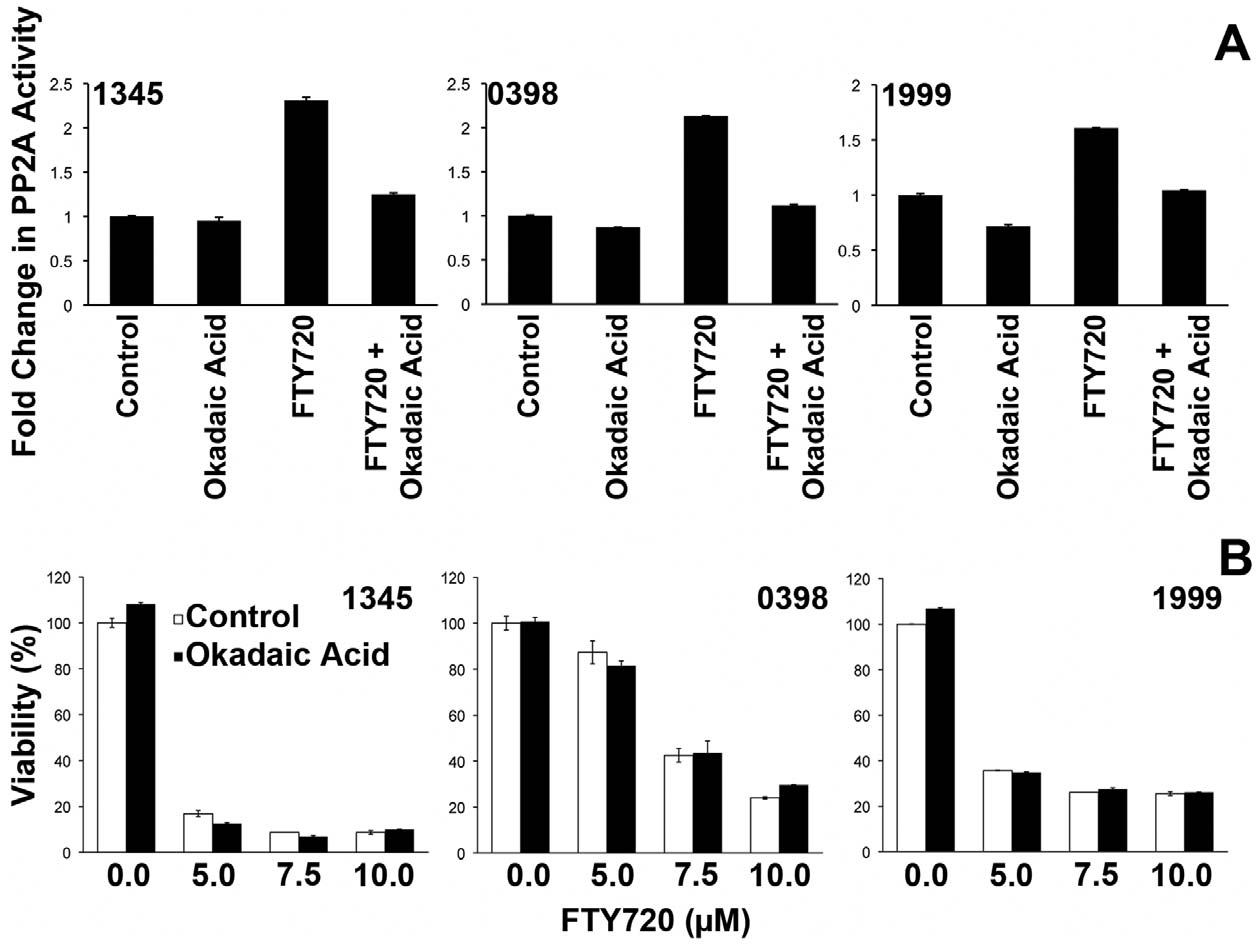
FTY720 reactivates PP2A but induces PP2A-independent cell death. (A) Xenografts were treated with 10 µM FTY720 for 4 hours with or without a 2 hour pre-incubation with 5 nM okadaic acid. Activation of PP2A was assessed as described in ‘Materials and Methods’. (B) All xenografts were treated with the indicated concentrations of FTY720 with or without a pre-incubation with 5 nM okadaic acid. Viability was assessed by flow cytometry using annexin V and propidium iodide staining. The mean ± SD of duplicate determinations are shown.

We considered the possibility that the Ph^−^ patient xenografts may have been resistant to FTY720 but *in vitro* culture demonstrated that 1345, 0398, 1999, ALL-3, ALL-55 and ALL-56 were all sensitive, with comparable IC_50_ values to ALL cell lines and patient samples examined previously, including the Ph^+^ sample 2070 [12] (Fig. 3). FTY720 induced activation of PP2A in all xenografts which was inhibited by 5 nM okadaic acid, a concentration previously shown to be optimal for specific inhibition of PP2A [8], However, FTY720-induced cell death was independent of PP2A activation (Fig. 4).

## Discussion

The main findings of this study were that FTY720 was ineffective in treating human Ph^−^ ALL xenografts whilst effective for Ph^+^ ALL in a clinically relevant mouse model of human ALL, implying that this drug is unlikely to be suitable for clinical trial development for Ph^−^ disease. Overall there were no significant reductions in ALL in Ph^−^ xenografts as a result of FTY720 treatment and in one xenograft we observed a clear and significant worsening of the disease. This lack of effect was observed not only when animals had advanced disease but also in an early disease model. The increased splenic infiltration observed in one xenograft could be due to FTY720-induced loss of S1P_1_ expression and retention of ALL cells in the spleen as S1P_1_ is required for lymphocyte egress from the splenic white pulp [20]. The reduced efficacy of FTY720 on inhibiting the infiltration of two of the three Ph^+^ ALL into the spleens is also consistent with an effect on ALL cell trafficking. However, the increased splenic disease in xenograft 0398 was not associated with significantly reduced disease in the blood or bone marrow suggesting that altered trafficking was not the only cause of increased ALL in the spleens of FTY720-treated animals. We found FTY720 to be efficacious in a human xenograft model of Ph^+^ ALL in the early treatment model, consistent with previous reports in mouse models of Ph^+^ ALL [11].

It is not clear why FTY720 was not effective *in vivo* for Ph^−^ disease but there are a couple of potential explanations. Firstly the bone marrow microenvironment has been reported to afford protection from the effects of a range of chemotherapeutic agents in a number of hematological malignancies including ALL [21]. Although the mechanism is not fully understood, stromal cells are known to provide factors that support ALL survival, activating pro-survival pathways such as the PI-3K/mTOR pathway [22], [23]. It is also possible that FTY720 simply did not attain sufficient concentrations in the animals to produce the cytotoxic effects in ALL cells that we observed *in vitro*. Pharmacokinetic studies of FTY720 performed in rats suggest that micromolar concentrations may not be achievable [19], [24], [25]. In our study, plasma FTY720 levels were approximately 200 nM and 400 nM when administered at 10 mg/kg/day by IP injection and 25 mg/kg/day oral, respectively. However, *in vivo* responses have been observed in a range of hematological malignancies where *in vitro* IC_50_ values were in the micromolar range (Table 1). The reasons for the discrepancies in effective drug concentrations *in vitro* and *in vivo* remain unclear. We have previously reported a similar situation with the mTOR inhibitor RAD001 in ALL [14], with IC_50_s in the low micromolar ranges, but *in vivo* responses observed at plasma concentrations ten-fold lower, and it is possible that effects on the microenvironment are contributing to the observed *in vivo* response. Lower concentrations of FTY720 appear to be required *in vivo* for Ph^+^ ALL than the *in vitro* studies would suggest. It is also possible that the previously reported accumulation of FTY720 in lymphoid tissues enhances its effective concentration on leukemia cells, relative to the concentration measured in plasma [26]. This increases the likelihood of FTY720 being useful in the setting of malignant disease. Although this may explain greater than expected *in vivo* activity, it would not explain the discrepancy between Ph^−^ and Ph^+^ ALL. While FTY720 has relatively low toxicity compared to standard chemotherapeutic agents, there are suggestions that long-term exposure produces macular degeneration [27] and conditions associated with increased vascular leak [28], as well as increased risk of viral infections and skin cancer [4]. FTY720 also induces transient bradycardia, which could explain the lethargy observed following injection [29].

In contrast to most previous studies, the ultimate mechanism of cell death *in vitro* in Ph^+^ and Ph^−^ ALL cells was both caspase- and PP2A-independent. Although PP2A was activated by FTY720, inhibition of PP2A activity using okadaic acid did not impact on cell death. This finding contrasts with many previous studies including those in hematological malignancies [8], [10], [11]. The major exception was a study in natural killer cell leukemia [13]. Despite reporting *in vitro* and *in vivo* efficacy, FTY720 was not found to activate PP2A and the PP2A inhibitor okadaic acid did not reverse FTY720-induced cell death. While our data is similar, in that cell death was PP2A independent, we did observe a significant activation of PP2A following FTY720 exposure. Our data also differs in that the mechanism of cell death was not caspase-dependent apoptosis.

Our previous *in vitro* studies also demonstrated the production of reactive oxygen species in response to FTY720, a finding that is in keeping with previous reports [9], [30]. In these studies blockade of ROS production by antioxidants partially reversed the cytotoxic effects of FTY720. In the study by Liao et al [13] it was suggested that ROS production down regulated the anti-apoptotic protein Mcl-1, the loss of which was required for cell death [13]. Similarly, we have shown that FTY720 reduced expression of Mcl-1, however we did not detect apoptosis as a result of FTY720 exposure [12].

Mcl-1 also inhibits the development of autophagy through inhibition of beclin-1 [31], [32]. Although we did not detect induction of beclin-1 expression with FTY720, reduced inhibition would be expected due to lower Mcl-1 protein levels thereby permitting the development of autophagy. Furthermore, autophagy induced by a sub-cytotoxic concentration of FTY720 partially counteracted the cytotoxic effects of agents such as vincristine supporting its cell protective role [12]. FTY720-induced autophagy is at least partly due to the induction of S1P signaling by FTY720-P [12], [19]. Brinkmann *et al* noted that FTY720-P peaked at more than triple the peak levels of the cytotoxic unphosphorylated FTY720 [19]. In our study, the non-cytotoxic phosphorylated derivative was found to be approximately 1.5 and 6 fold higher than FTY720 in animals receiving FTY720 by the IP and oral route respectively. It therefore appears that the induction of autophagy may offer protection, which may have played a role in our animal experiments, potentially explaining the outcomes observed for Ph^−^ disease.

Our observation that a drug with excellent *in vitro* efficacy did not universally translate into positive effects in the *in vivo* setting was unexpected. The disparity between the *in vivo* responses of Ph^−^ ALL samples to FTY720, despite good *in vitro* efficacy currently lacks an adequate explanation, but highlights the importance of conducting well-designed pre-clinical studies prior to initiating clinical trials. In conclusion, we present here the first report of FTY720, in a relevant human xenograft model, demonstrating differential efficacy in Ph^+^ versus Ph^−^ B-lineage ALL, a finding which questions the utility of further testing of FTY720 in the latter form of the disease.

## Supporting Information

Table S1 **Clinical information.**
(DOC)
