# Correction: Disparate In Vivo Efficacy of FTY720 in Xenograft Models of Philadelphia Positive and Negative B-lineage Acute Lymphoblastic Leukemia

**DOI:** 10.1371/annotation/d7342840-682b-4ee2-bb41-7b41aa5dedf7

**Published:** 2012-08-09

**Authors:** Craig T. Wallington-Beddoe, Anthony S. Don, John Hewson, Qiao Qiao, Rachael A. Papa, Richard B. Lock, Kenneth F. Bradstock, Linda J. Bendall

There is an error in Figure 2. A correct image for Figure 2 can be seen here: 

**Figure:**
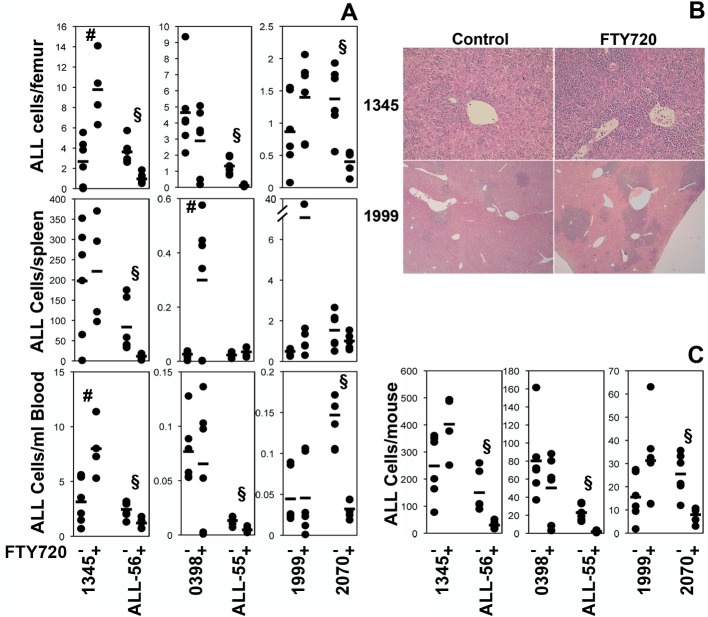


[^] 

