# Supplementary material for: Disparate In Vivo Efficacy of FTY720 in Xenograft Models of Philadelphia Positive and Negative B-lineage Acute Lymphoblastic Leukemia
Source: PLoS One. 2012 May 3;7(5):e36429. doi: 10.1371/journal.pone.0036429 (PMC3343039; doi:10.1371/journal.pone.0036429)
Supplement: Table S1 — Clinical information. (DOC) [file pone.0036429.s001.doc]

**Table S1.** **Clinical information**

| **Patient ID** | **Sex/Age** | **Source** | **% Blasts** | **Immuno-phenotype** | **Cytogenetics** |
| --- | --- | --- | --- | --- | --- |
| **ALL-3** | F/12 | BM | > 85 | CD10^-^CD19^+^ | 46 XX, del(11;q23) |
| **ALL-55** | M/14 | BM | >90 | CD10^+^ CD34^+^CD19^+^ | t(9;22)(q34;q11.2)* |
| **ALL-56** | M/10 | BM | >90 | CD10^+^ CD34^+^CD19^+^ | 46 XY t(9;22)(q34;q11.2)[6]/46idem, der(16)t(1;16)(q21;q12)[6]/46 idem +10,der(16)t(1;16) |
| **1345** | F/5 | BM | 95 | CD10^+^CD19^+^ | 45 XX dup(1)(q42 q25), del(3)(q21), -9, del(9)(p22), t(18;20)(q21q13.1) |
| **1999** | F/14 | BM | 94 | CD10^+^CD19^+^ CD34^-^ | 46 XX |
| **0398** | M/15 | BM | 96 | CD10^-^CD19^+^ CD34^+^ | 46 XY add(3)(q29) t(14:19)(q32p13) |
| **2070** | M/65 | BM | N/A | CD10^+^ CD34^+^CD19^+^ | 45 XY t(9;22) (q34;q11.2) del(9) (p21) |

N/A - not available. *Determined by PCR analysis.
